# Homologous illegitimate random integration of foreign DNA into the X chromosome of a transgenic mouse line

**DOI:** 10.1186/1471-2199-11-58

**Published:** 2010-08-13

**Authors:** Bowen Yan, Defa Li, Kemian Gou

**Affiliations:** 1 State Key Laboratory for Agrobiotechnology, College of Biological Sciences, China Agricultural University, Beijing 100193, China; 2 College of Animal Sciences & Technologies, China Agricultural University, Beijing 100193, China

## Abstract

**Background:**

It is not clear how foreign DNA molecules insert into the host genome. Recently, we have produced transgenic mice to investigate the role of the *fad2 *gene in the conversion of oleic acid to linoleic acid. Here we describe an integration mechanism of fad2 transgene by homologous illegitimate random integration.

**Results:**

We confirmed that one *fad2 *line had a sole integration site on the X chromosome according to the inheritance patterns. Mapping of insertion sequences with thermal asymmetric interlaced and conventional PCR revealed that the foreign DNA was inserted into the XC1 region of the X chromosome by a homologous illegitimate replacement of an entire 45,556-bp endogenous genomic region, including the ovarian granulosa cell tumourigenesis-4 allele. For 5' and 3' junction sequences, there were very short (3-7 bp) common sequences in the AT-rich domains, which may mediate the recognition of the homologous arms between the transgene and the host genome. In addition, analysis of gene transcription indicated that the transgene was expressed in all tested *fad2 *tissues and that its transcription level in homozygous female tissues was about twice as high as in the heterozygous female (p < 0.05).

**Conclusions:**

Taken together, the results indicated that the foreign *fad2 *behaved like an X-linked gene and that foreign DNA molecules were inserted into the eukaryotic genome through a homologous illegitimate random integration.

## Background

Direct microinjection of foreign DNA into the pronucleus of fertilised zygotes is a conventional method to generate transgenic animals, whereas the exact integration site and the number of copies of the transgene are random and unpredictable [1,2]. Previous studies involving animal transgenesis indicate that the linear DNA molecules injected into the pronucleus undergo rapid circularisation followed by random linearisation and concatemer formation by homologous recombination before integration into the host genome [3-5]. It was thought that the foreign DNA concatemers would be finally inserted into the host DNA randomly through imperfect sequence recognition via heterologous recombination followed by cellular DNA repair activity [2,6]. Until now, it was not clear how foreign DNA molecules insert into the host genome. A few studies have unravelled some of the mystery of random integration and indicated that the integration site of foreign DNA is not totally random [6,7]. More detailed analyses of the integration sites revealed some interesting trends. For instance, a review of 35 different insertion mutants generated in transgenic mouse lines revealed that some chromosomes, such as chromosome 10 and 6, are selected more often for illegitimate integration than others [8]. Intrinsic DNA structures such as bent DNA elements could be a major determinant in chromosomal illegitimate recombination because their structure can provide a preferential donor site for the integration [9,10]. In addition, short identical sequences of 1 to 3 nucleotides have been found at the genome-transgene junctions [11]. These integration sites are usually associated with the consensus sequence for topoisomerase-I cleavage sites [11,12].

Recently, we have successfully used standard pronuclear microinjection to produce transgenic mice integrated with the *fad2 *gene from the cotton plant, encoding fatty acid desaturase-2. Those transgenic mice were used to study the role of FAD2 in the conversion of oleic acid to linoleic acid. In the present study, using the transgene inheritance pattern of F1 progeny, we showed that one of the transgenic lines had only one integration site on the X chromosome. Thermal asymmetric interlaced PCR (TAIL-PCR) [13,14] was used to identify the transgene-chromosome junction in mice previously [15-17]. To investigate the exact insertion site on the X chromosome, this method was also employed to map the 3' chromosomal boundaries of the integration site in *fad2 *mice. We successfully defined the 3' integration site by TAIL-PCR and the 5' integration site by conventional PCR. Based on the sequence data of both junctions, the mechanism of the homologous illegitimate random integration of the foreign DNA in transgenic animals was also analysed. Finally, the transcription characteristics of the X-linked *fad2 *were investigated further.

## Results

### Analysis of transgene inheritance

**Figure 1:**
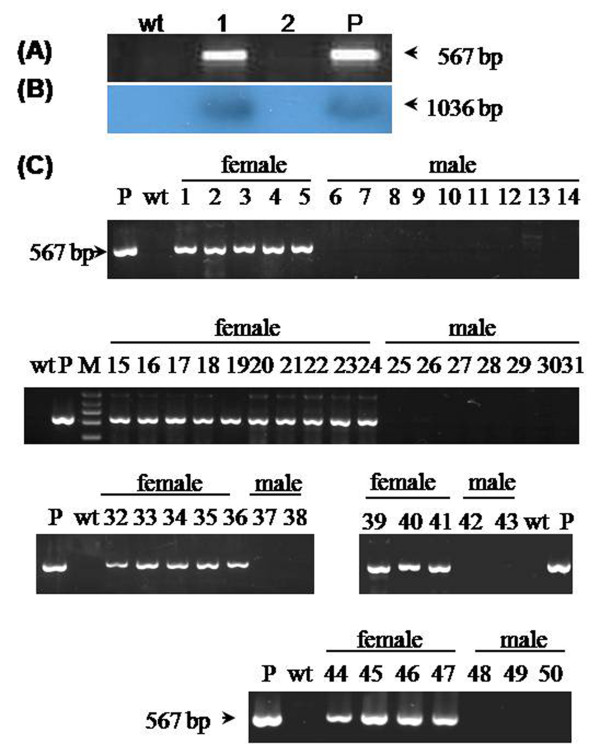
**Transgene analysis**. (A) PCR and (B) Southern blot analysis of total DNA from wild-type C57 (wt), mouse-1 (lane 1) and mouse-2 (lane 2) shows that mouse-1 was *fad2 *transgenic, whereas mouse-2 was nontransgenic. P, the mixture of *fad2 *plasmid and wt genomic DNA (A) or the 1036-bp fragments of *fad2 *transgene digested by *EcoR *I and *Sca *I enzymes together (B). (C) PCR analysis of total DNA from 50 heterogeneous F1 mice produced by natural mating of the C57 females and mouse-1 (male) show that all 27 F1 females (mice nos. 1-5, 15-24, 32-36, 39-41, and 44-47) were transgenic, whereas all 23 F1 males (mice nos. 6-14, 25-31, 37-38, 42-43, and 48-50) were nontransgenic. M, DNA marker. P, the mixture of *fad2 *plasmid and wt genomic DNA.

PCR (Figure 1A) and Southern blotting analysis (Figure 1B) demonstrated that a male mouse (mouse-1) integrated the foreign gene. PCR analysis of its F1 offspring produced by seven C57 females showed that all 23 heterogeneous males were nontransgenic, whereas all 27 heterogeneous females were transgenic (Figure 1C). The results indicated that the *fad2 *transgene integrated into the X chromosome.

### Identity of the 3' integration site

**Figure 2:**
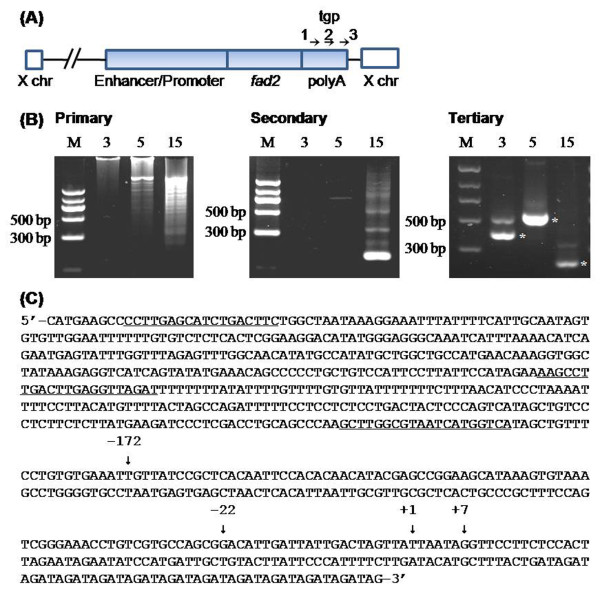
**TAIL-PCR analysis of the 3' integration site**. (A) Schematic diagram of the transgenic construct indicating the positions of three transgene primers (tgp) used, along with an arbitrary degenerate primer. (B) Gel analysis of TAIL-PCR products amplified from the *fad2 *mice. Total DNA from three *fad2 *mice (nos. 3, 5, and 15) was used for TAIL-PCR, and the specific amplified fragments (*) in each sample from the tertiary amplification were sequenced directly. (C) Nucleotide sequence of transgenic forward primers (underlined) tgp1 (-627 to -610), tgp2 (-381 to -360), and tgp3 (-206 to -187) and the 3' integration region in *fad2 *mice. The sequence from -172 to the 3' end was determined by direct sequencing. The sequencing results indicate that nucleotides from -172 to +7 were 100% identical to the transgene sequences and that the nucleotides from +1 to the 3' end were the same as the downstream sequence of the 87,507,732nd nucleotide of the XC1 region of the X chromosome. Seven nucleotides, from +1 to +7 (5'-TTAATAG-3'), were shared by the transgene and the X chromosome as a very short homologous arm. Furthermore, the sequences from -172 to -22 and -22 to +7, corresponding to the 3' end and the 5' initial transgene sequences, indicate that the foreign DNA molecules integrated as a head-to-tail array.

To determine the integration site of the foreign DNA on the X chromosome, TAIL-PCR was used to define the 3' chromosomal boundaries of the transgene (Figure 2A). Each PCR fragment, amplified by the tertiary cycling from the samples of three mouse-1 progeny, was purified and sequenced directly (Figure 2B). Sequencing and BLAST search results in the *Mus musculus *(C57BL/6J) genome showed that the mouse-15 fragments were derived from the transgene itself, whereas PCR products of mouse-3 and mouse-5 contained both the foreign DNA and the X flanking sequences (Figure 2C). Homology analysis indicated that the foreign gene was integrated into the XC1 region and that the junction site was located at base 87,507,732.

Junction sequence analysis also showed that there was an additional 29-bp fragment (Figure 2C, from -22 to +7) between the 3' end of the transgene and the X flanking sequence. This 29-bp sequence was 100% identical to the initial 5'-end of the foreign DNA in which the last seven nucleotides (5'-ttaatag-3', Figure 2C, from +1 to +7) were also shared by the X chromosome sequence. Additional PCR amplification using the specific primers that spanned the above junction sequence confirmed that the 3' integration site (data not shown) was successfully mapped. These results suggest that the transgene integration was mediated by the seven common nucleotides.

### Identity of heterozygotes

**Figure 3:**
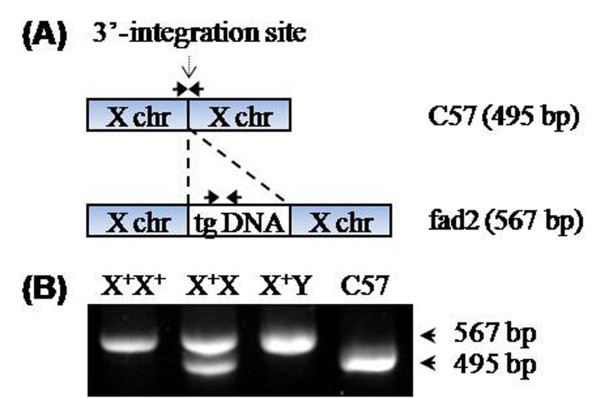
**Identity of homozygous or heterozygous fad2 mice**. (A) Schematic diagram of the primer positions indicating the fragments spanning the 3' integration site in wild-type C57 or transgene-specific fragments in *fad2 *mice. (B) Site-specific primers used to identify *fad2 *homozygotes and heterozygotes. C57 samples had the 495-bp product only, *fad2 *males (X^+^Y) and homozygous females (X^+^X^+^) had the 567-bp transgene products only, and the heterozygous *fad2 *females (X^+^X) had both bands together. X^+ ^and X represent the X chromosome integrated with fad2 transgene and wild-type X chromosome, respectively.

Genotypes were determined by PCR amplification using transgene-specific (567-bp) and genome-specific primer (495-bp) sets concurrently. As shown in Figure 3, amplification of the heterozygous transgenic DNA in the X^+^X female sample resulted in two bands, 567 bp and 495 bp. C57 DNA yielded only a single 495-bp band, and homozygous transgenic DNA in the X^+^X^+ ^female or the X^+^Y male yielded only a 567-bp band.

### Identity of the 5' integration site

**Figure 4:**
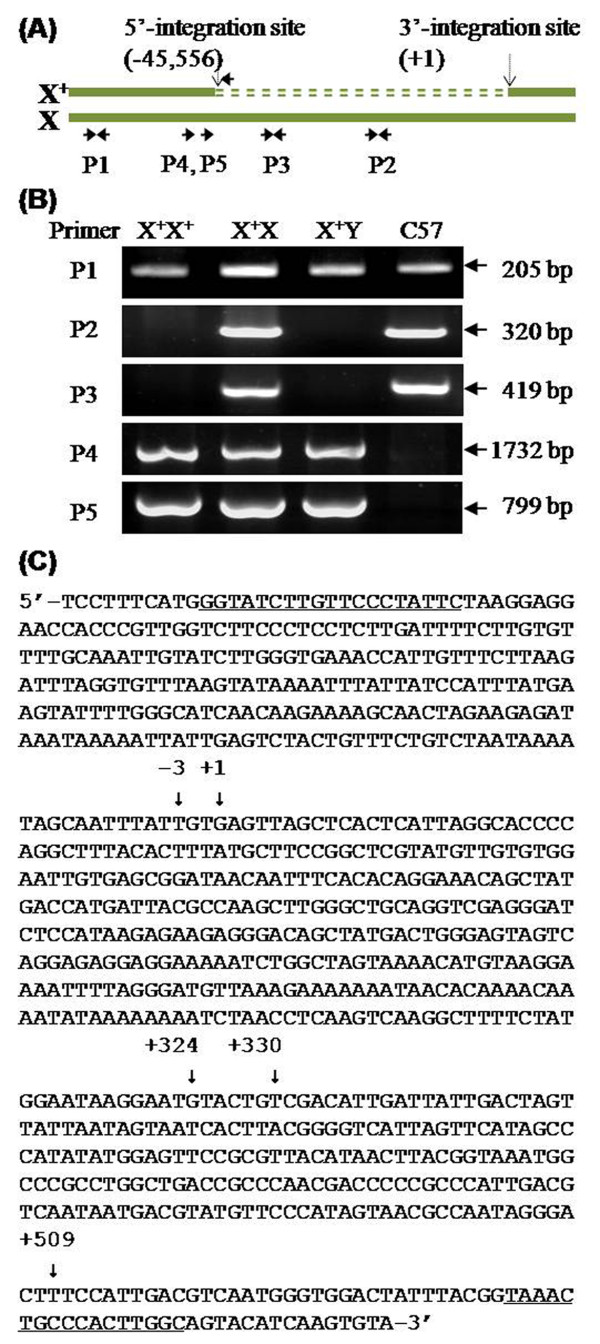
**PCR analysis of the 5' integration site**. (A) Schematic diagram of the approximate positions of five primer sets used for PCR analysis of the 5' integration site. P1 (-54,088 to -53,884), P2 (-29,872 to -29,553), and P3 (-44,562 to -44,144) primer pairs were designed according to the sequence upstream of nucleotide 87,507,732 (+1), whereas the forward primers of P4 (from -46,680) and P5 (from -45,797) were designed corresponding to the X sequence. The reverse primer (5'-gccaagtgggcagttta-3') corresponded to the CMV enhancer sequence included in the transgene construct. X^+ ^represents the transgenic X. The double-dashed line represents the foreign DNA molecules. (B) Gel analysis of the PCR products amplified from the *fad2 *or C57 mice. The 205-bp fragments using P1 primers were successfully amplified in X^+^X^+^, X^+^X, X^+^Y, or C57 samples. The 320-bp fragments using P2 primers and the 419-bp fragments using P3 primers were only amplified in the X^+^X and C57, not in X^+^X^+ ^or X^+^Y, samples. The 1732-bp fragment amplified by P4 primers and the 799-bp fragment amplified using P5 primers were present in all *fad2*, but not C57, mouse samples, and they were sequenced directly. (C) Nucleotide sequence of the P5 primer pair (underlined) and the 5' integration region in *fad2 *mice. Sequences from the 5' initial nucleotide to +509 were determined by direct sequencing. The partial sequence from the 5' initial nucleotide to -1 was 100% identical to the region upstream of the 87,462,177th nucleotide of the XC1 region of the X chromosome. The sequence from -3 to +509 showed 100% identity to the transgene sequence. A 5'-TGT-3' sequence (-3 to -1) was shared by the transgene and the X chromosome as a very short homologous arm. Transgenic sequences were divided into two classes based on the presence of five additional nucleotides (TACTG). The sequence from -3 to +324 was 100% identical to the foreign complementary sequence (from 3580 to 3259). The sequence from +330 to the 3' end was 100% identical to the 5' initial sequence of the transgene.

Based on the 3' integration site at 87,507,732, several primer sets corresponding to its known upstream sequence (GenBank accession no. NT_039706.7) were designed to map the 5' integration position. Among them, the results of three amplifications are shown in Figure 4. P1 primers, corresponding to the -54,088 to -53,884 region, successfully amplified the 205-bp fragment in all of the X^+^X^+^, X^+^X, X^+^Y and C57 samples. P2 and P3 primers, corresponding to regions -29,872 to -29,553 and -44,562 to -44,144, respectively, only amplified the 320-bp and 419-bp fragments, respectively, in X^+^X and C57, but not in the X^+^X^+ ^or X^+^Y samples (Figure 4B). These results indicate that the 5'-integration site was within the -53, 884 to -44,562 region.

Further PCR analysis was performed using several additional primer sets consisting of the forward primers, corresponding to X loci from -53, 884 to -44,562, and the common reverse primer, corresponding to the CMV enhancer sequence included in the transgene construct. Among them, results of two amplifications are shown in Figure 4. P4, from -46,680, or P5, from -45,797, successfully produced the 1732-bp and 799-bp bands, respectively, in all *fad2 *samples, but not in the C57 samples. Sequencing and homology analysis of two fragments showed that the resulting sequence could be divided into three parts. The 3'-end sequence was 100% identical to the initial sequence of the transgenic molecules. The middle sequence, including 327 nucleotides (from -3 to +324 in Figure 4C), showed 100% identity with the transgenic complementary sequence from 3580 to 3259 that linked the 3'-end sequence with five additional nucleotides. The 5'-end sequence was 100% identical to the XC1 upstream sequence at position 87,462,177, in which three common nucleotides from -3 to -1 (5'-TGT-3') were shared by the transgene and the X chromosome (Figure 4C).

**Figure 5:**
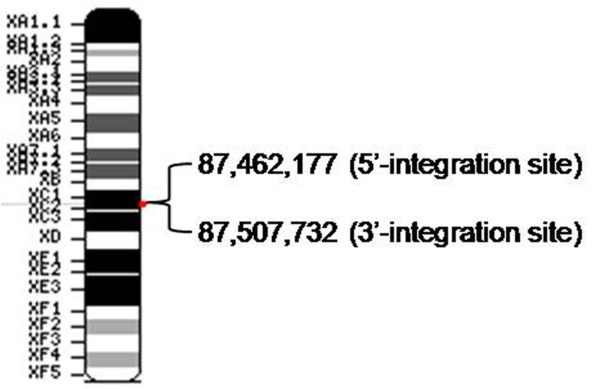
**Transgene mapping on the X chromosome**. The map of mouse chromosome X shows that the entire 45,556-bp region from 87,462,177 to 87,507,732 of XC1 was replaced by the foreign DNA during the process of random integration.

**Figure 6:**
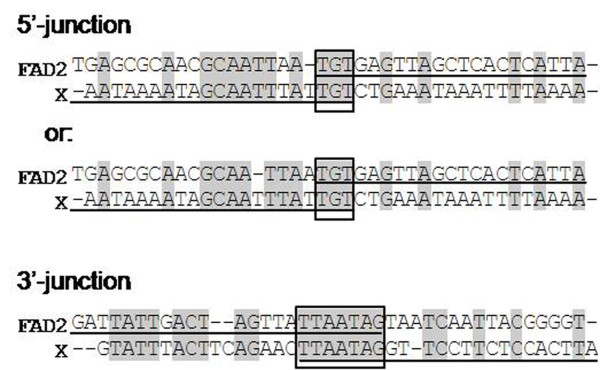
**Analysis of the sequences surrounding the junction sites**. The junction sequences present in the *fad2 *mice are underlined. Identical nucleotides in *fad2 *and X chromosome are indicated (grey shading). Three or seven identical nucleotides (boxed with grey shading) existed in the AT-rich domain, presumably corresponding to the common sequence of the recombination of transgene and host genome.

Based on the 5' and 3' junction mapping results, we concluded that the entire 45,556-bp genomic region (from 87,462,177 to 87,507,732, in the XC1 region of chromosome X) was successful replaced by the foreign DNA molecules during the process of random integration (Figure 5). For each junction, a very short homologous arm (3-7 bp) in the AT-rich domain presumably mediated the recombination of the transgene and the host genome (Figure 6).

### Transcription analysis

**Figure 7:**
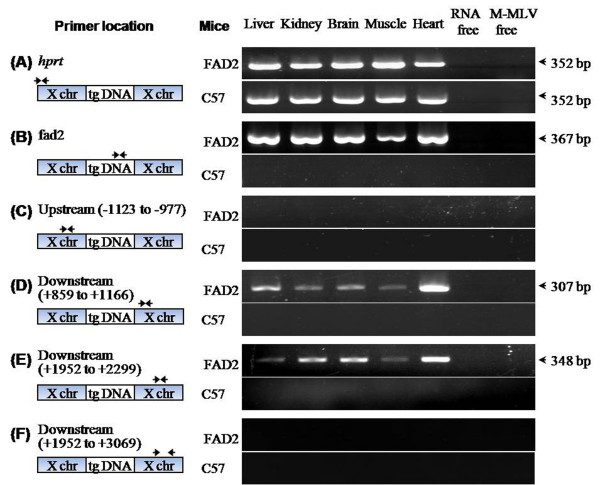
**RT-PCR analysis of the transgene and the X-linked sequences**. Total RNA from liver, kidney, brain, muscle, and heart tissues of the C57 or homozygous *fad2 *females was analysed using the different sets of primers. (A) The X-linked *Hprt *gene (normalisation control) showed normal expression in all samples from the *fad2 *and C57 mice. (B) Transgene specific primers revealed that the *fad2 *gene was expressed only in the transgenic mouse, not in the C57 mouse. (C-F) Transcripts of the region surrounding the X insertion site (from -1123 of the 5' integration to +3069 of the 3' integration site) were not detected in any C57 tissue. In *fad2 *mice, the examined upstream sequences (-1123 to -977) did not amplify (C), whereas RT-PCR fragments (D-E) of the downstream regions from +859 to +2299 were amplified successfully. The distal downstream region (from +1952 to +3069) was not detected in any of the examined transgenic tissues (F). Amplification without reverse transcriptase in each samples showed no contamination.

PCR and sequencing analysis using primer pairs spanning the 5' and 3' transgenic sequences showed multiple copies of the transgene inserted into the same site of chromosome X (data not shown). To assess the transcriptional potential of *fad2*, total RNA from liver, kidney, brain, muscle, and heart tissues was analysed by RT-PCR. As shown in Figure 7, the amplification results from five primer sets corresponding to different regions revealed that no transcriptional signals were detected in the upstream region (-1123 to -977) of the 5' integration site or the distal downstream region (+859 to +3069) of the 3' integration site in the C57 samples, while *fad2 *was expressed in all of the transgenic tissues (Figure 7B). In addition, the transcriptional signals of the 3' flanking sequences in the *fad2 *mice, which extended at least 2299 but no more than 3069 nucleotides past the 3' integration site, were detected by RT-PCR (Figure 7D-F).

### Transcription level of the X-linked transgene

**Figure 8:**
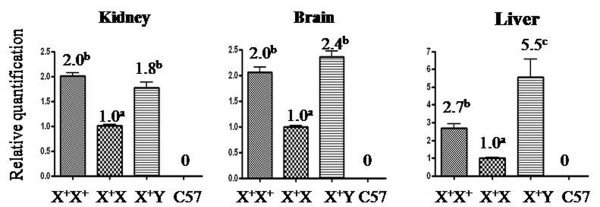
**Relative expression of the X-linked transgene**. Sets of primers corresponding to *fad2 *or *Gapdh *were used for real-time RT-PCR analysis of the samples of transgenic or C57 kidney, brain, and liver. Expression data of *fad2 *were normalised to *Gapdh *expression and presented as the mean relative quantity (compared with X^+^X), with error bars representing the SEM. Student's *t*-test was used to calculate *P *values. Values with different superscripts were significantly different within same tissues (*P *< 0.05).

To gauge the transcription level of the X-linked *fad2 *in females, we measured the expression of the foreign gene. Kidney, brain, and liver tissues from the X^+^X^+^, X^+^X, C57 females, or X^+^Y males were analysed concurrently for reference. The real-time RT-PCR analysis showed that the foreign gene was expressed in all transgenic samples from the *fad2 *females or males and that the transcription level in the X^+^X^+ ^females or X^+^Y males was about twice as high as in the X^+^X females (p < 0.05, Figure 8). The results suggest that the foreign *fad2 *behaved like an X-linked gene.

## Discussion

Approximately 5-10% of the random DNA insertion events in transgenic animals are associated with recessive mutations or viable phenotypic alterations [4,18]. Integrated DNA may affect the endogenous genetic locus and result in inactivation of a given gene [19,20]. In some cases, transgene integration has been associated with host genome rearrangements, including duplications [21], translocation [22], and deletions [23-25]. The length of the deleted genome segments might be from 2-3 kb [23] to 22 kb [25]. In the current study, we successfully identified a foreign gene homologous inserted into the XC1 region by deletion of the entire 45,556-bp region of the endogenous DNA. Although the DNA deletion took place, all *fad2 *mice appeared normal, without any apparent lesions, and exhibited normal physiological activities and fertility. Sequence analysis revealed that no putative genes were positioned in the deleted 45,556-bp region in which one Gct4 phenotype allele of ovarian granulosa cell tumourigenesis 4 was involved (MGI ID: 98356). Previous studies involving juvenile granulosa cell tumours indicate that its susceptibility is an inherited, polygenic trait and that the X-linked *Gct4 *allele in the SJL mouse strain cause high-frequency, juvenile-type granulosa cell tumour development in females [26,27]. The *fad2 *gene inserted into the mouse genome could serve both as a mutagen and as a molecular tag to study the role of the *Gct4 *allele in juvenile-type granulosa cell tumour.

**Figure 9:**
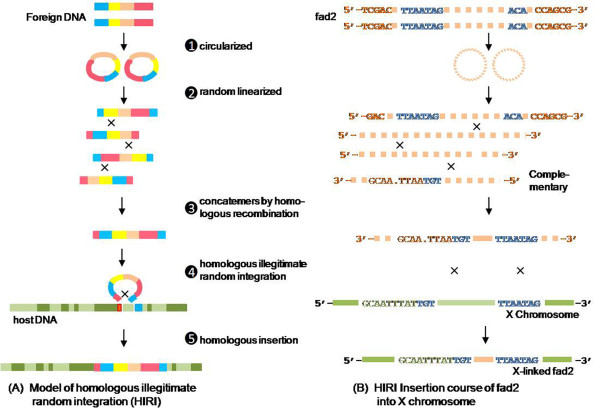
**Random integration mechanism of foreign DNA by homologous illegitimate random integration and course of fad2 inserted into the X chromosome**. (A) Identical foreign DNA injected into the nucleus of a eukaryotic cell is circularised and randomly cleaved by the endogenous restriction enzyme(s). It generates tandem concatemers by homologous recombination (steps 1-3, adapted from reference [2]). The concatemers are inserted into the host genome, mediated by homologous illegitimate random integration (HIRI), which depends on several identical nucleotide sequences (in red or blue) in the AT-rich domains on both sides, which serve as anchors for the two DNAs. Consequently, a host DNA region is replaced by the foreign DNA. During DNA replication, the repair mechanism of the cell induces foreign DNA integration into the host genome (steps 4-5). (B) *Fad2 *transgenic concatemers consist of a ≥322-bp complementary DNA and multiple copies inserted in a head-to-tail array by homologous recombination. During the HIRI process, 5'-TGT-3' and 5'-TTAATAG-3' (capital letters in blue) in the AT-rich domain, which exist in both foreign DNA and the X chromosome, mediate the illegitimate recombination of the transgene and the X chromosome as the homologous arms. When the homologous foreign DNA is inserted into the X chromosome, all of the 45,556 target nucleotides on the X chromosome are replaced by the foreign DNA.

It is thought that linear DNA molecules injected into the pronucleus undergo rapid circularisation followed by random linearisation and concatemers formation by homologous recombination (Figure 9A, steps 1-3). Moreover, the injected molecules could then reconstitute and, most frequently, integrate as a head-to-tail array [2,3,5,28,29], with a minor modification of nucleotide deletion or insertion at some ends [7,11,30]. In the present study, the multiple copies of the *fad2 *transgene molecules lost the 5' initial GC dinucleotide, which was subsequently integrated into the X chromosome in a head-to-tail manner (Figure 2C). In addition, a head-to-head fusion also occurred in the transgene, whereby one strand joined its partial complementary strand with five additional nucleotides at the same insertion site (Figure 4C, Figure 9B).

As far as we know, it is not clear exactly how the concatemers insert into the host genome. It was previously reported that the foreign histocompatibility class II E_α _gene, injected into pronuclei, was homologous targeted into the transgenic mouse genome [31]. In most cases, it is predicted that the foreign DNA concatemers would finally integrate into the host genome randomly through imperfect sequence recognition via heterologous recombination [2,21]. In the current study, it is worth noting that the 3-bp common sequence at the 5' junction and the 7-bp common sequence at the 3' junction were shared by the X chromosome and foreign transgenic molecules. Furthermore, the homologous nucleotides in the 5' junction would facilitate the recognition of two DNAs besides the 3-bp common sequence (Figure 6, Figure 9B). Such small regions of identity (3-5 bp) have been observed in previous studies of non-homologous recombination involving mouse transgenesis [11,29]. This suggests that the successful insertion of the transgene is mediated by these common sequences on both sides, which serve as anchors for the homologous illegitimate random integration (HIRI) but not heterologous recombination (Figure 9). Because the homologous arms are very short and consist only of several identical nucleotides, they can be easily positioned at various regions of the host genome. Therefore, it appears that the foreign DNA is randomly integrated into the host genome and the integration sites are unpredictable in transgenic animals [1,2]. After homologous recognition between the two DNA molecules, the insertion of a foreign DNA molecule into a genome is mediated by the DNA repair mechanisms of the cell [2,6].

In addition to the common sequence, a palindromic sequence of 5'-ATTAAT-3' in both 5' and 3' junctions was found in the *fad2 *sequence, but not in the X sequence (Figure 6). This sequence motif is required to induce a localised conversion in *Streptococcus pneumoniae *transformation [32]. The *Ase *I restriction endonuclease can recognise and cleave the sequence between the TT dinucleotide [33]. At this moment, we are not sure if an endonuclease plays a role in the HIRI process.

In mechanistic studies of natural transformation in prokaryotes, de Vries & Wackernagel reported that short stretches of sequence identity (3-8 bp) between the kanamycin-resistant nptII^+ ^gene and the recipient DNA of transformed Acinetobacter facilitated the integration efficiency of foreign DNA into the prokaryotic genome by homology-facilitated illegitimate recombination using homologous regions ranging in length from 1 kb to 183 bp [34]. Simultaneously, a consistent integration mechanism, mediated by one-side homologous substrates and containing identical 4-10 nucleotide sequences between the donor and recipient DNA, was also observed in the human pathogen *Streptococcus pneumoniae *transformed by recombinant lambda bacteriophage [35]. In addition, in *Pseudomonas stutzeri *a similar phenomenon was observed, in which part of the short anchor segments recombined into the host genome [36]. In eukaryotes, events of illegitimate integration whose homologous sequences are very short (≤5 bp) are also found in mammalian cells [7,37], transgenic embryos [11], and mice [29]. Taken together, our observations suggest that the HIRI process is present in all organisms undergoing transformation/transgenesis (Figure 9A).

Interestingly, although the HIRI process in prokaryotes usually happens within segments of high GC content [34-36], our results reveal that it occurred in the AT-rich, not GC-rich, region between the two DNAs in eukaryotes (Figure 6). Similar results have been found in the transgenic embryo [11]. A systematic analysis of multiple illegitimate integration sites in somatic cells has found that in 93% of cases, these sites are only 10 bp away from a potential topoisomerase I cleavage site. The association of topoisomerase I sites with runs of purines and AT-rich regions with the site of integration is also significant [12]. Purine tracts can adopt non-B-DNA conformations, which may be able to recombine; these sequences are found in the centromeres and may promote recombination of the satellite DNA [6]. These observations suggest that AT-rich regions might be involved in the HIRI process.

Although in murine transformed 3T6 cells the poly (A) signal of the rabbit β-globin can direct efficient termination of polyomavirus DNA transcription through RNA polymerase II [38], in HeLa cells, in some cases, transcription of the foreign gene does not terminate thoroughly by the terminal signal of the 537-bp poly (A) full sequences [39]. The nucleotide sequence of the 3' flanking region of the rabbit β-globin gene was transcribed 2447 bp past the poly (A) site. The transcription level in the M13 bacteriophage vector gradually declines, under control of its own enhancer and promoter [40]. In *fad2 *mice, the transgene was expressed efficiently under the driven of the CMV enhancer and β-*actin *promoter (Figure 7, Figure 8) and its transcription had not been terminated thoroughly by the terminal signal of the rabbit β-globin gene poly (A) yet. RT-PCR results revealed that the 2.3 kb flanking regions post the 3' integration site was transcribed within all examined *fad2 *tissues whereas these genomic regions kept silence in wild type (Figure 7D, 7E). Consistent with the previous study [40], these transcription level might gradually declines and the transcripts of the 3.0-kb distal flanking regions of the 3' integration site had not been detected within all examined *fad2 *tissues (Figure 7F).

In mammals, dosage compensation takes place by silencing one of the two X chromosomes in female cells to achieve transcriptional balance with the XY male [41,42]. Transgenes carried on the X chromosome occasionally either escape the normal X-inactivation process [43] or behave like an X-linked gene [44,45]. The cloning and characterisation of the host sequences flanking these inserts may contribute to our understanding of the molecular control mechanisms of chromosome pairing [46,47] and mammalian X-inactivation [44,45]. As an X-linked transgene, the relative expression of *fad2 *was also examined in transgenic somatic tissues. The transgene expression behaved in a fashion similar to silence one of the two X chromosomes in female cells, at least within the tested female tissues. That was, only 50% of X^+^X cells expressed the fad2 gene, whereas all X^+^X^+ ^female cells or X^+^Y male cells expressed the transgene. Subsequently, the relative amount expression of fad2 gene in the X^+^X organs was lower and estimated an approximately 50% amount of the X^+^X^+ ^female or X^+^Y male cells. These results suggest that the XC1 region (from 87,462,177 to 87,507,732) is a locus amenable to the normal X-linked expression of foreign genes and can be used as a molecular tag to study the mechanism of X inactivation.

## Conclusions

An X-linked transgenic mouse line is identified firstly in the current study. The real-time RT-PCR analysis indicates that the foreign *fad2 *gene is expressed in all transgenic samples from the *fad2 *females and that the transcription level in the homozygous females is about twice as high as in the heterozygous females. That is, the transgene expression behaves in a fashion similar to silence one of the two X chromosomes in female cells, at least within the tested female tissues.

We successfully map the sequences of both sides of transgene-chromosome in fad2 transgenic mice and identify that the 5' and 3' integration sites are located at base 87,462,177 and 87,507,732, respectively. PCR analysis reveals that the entire 45,556-bp genome in the XC1 region of chromosome X is deleted by the foreign DNA molecules during the process of random integration. The deleted 45,556-bp endogenous genomic region includes the ovarian granulosa cell tumourigenesis-4 allele.

For each junction sequence, a very short homologous arm (3-7 bp) in the AT-rich domain, for instance, TGT within the 5' junction and TTAATAG within the 3' junction, exists in both foreign *fad2 *gene and the X chromosome and presumably mediates the illegitimate recombination of two DNAs as the homologous arms. Based on the 5' and 3' junction mapping results, we predict that the foreign DNA insert into the host genome through a homologous illegitimate random integration (HIRI), which depends on several identical nucleotide sequences in the AT-rich domains on both sides.

## Methods

### Animals

All animals were maintained in a light-controlled room at 22°C. All animal procedures were approved by the Committee for Experimental Animals of our university.

### Transgene construct

An 1182-bp DNA sequence of the cotton *fad2 *open reading frame (GenBank accession No. X97016) was optimised and synthesised (Invitrogen) with a modification of codon usage for easier expression in mammals. The expression vector for microinjection contained the *fad2 *gene driven by the cytomegalovirus (CMV) enhancer and the chicken β-*actin *promoter, as well as the rabbit β-globin poly (A) sequence at the 3' end. Transgenic mice were produced by pronuclear microinjection with the linear 3.6-kb transgenic cassettes digested by *Sal *I and *Bam*H I.

### Transgene detection by nucleic acid analysis

Genomic DNA was extracted from the tails of pups (3-4 weeks old) utilising the standard phenol-chloroform method, as previously described [1], and dissolved in TE buffer for nucleic acid analysis. The presence of the transgene was assayed by PCR amplification using transgene-specific primers (F: 5'-tacatcagcgacacaggcatc-3'; R: 5'-gtatttgtgagccagggcatt-3'). The amplified product was 567 bp long and spanned the *fad2 *gene sequence. PCR reactions contained 0.5-1.0 μg of genomic DNA, 0.2 mM dNTPs, 0.4 μM of primers, and 1 unit of *Taq *polymerase (Invitrogen) in 25 μl of 1 × reaction buffer. The reactions were performed at 94°C for 5 min; 35 cycles of 94°C for 30 sec, 58°C for 30 sec, and 72°C for 40 sec; and 72°C for 8 min.

For Southern blotting analysis, 10 μg of the genomic DNA was digested with *EcoR *I + *Sca *I for 12-16 hours, separated on 0.7% agarose gels overnight and blotted to Hybond-N+ membranes (Amersham, UK). The 1036-bp fragments from the pFAD2 plasmid produced by *EcoR *I + *Sca *I digestion were labelled with α-^32^P-dCTP (FuRui, China) using the Rediprime™II labelling kit (Amersham). The membranes were subsequently hybridised with the labelled probe according to standard protocols and exposed to X-ray film (Fuji Photo Film, Japan) for 24-48 hours at -80°C with an intensifying screen to obtain an autoradiograph image.

### TAIL-PCR analysis of the 3' integration site

Three transgene specific primers (tgp1: 5'-ccttgagcatctgacttct-3'; tgp2: 5'-aagccttgacttgaggttagat-3'; tgp3: 5'-gcttggcgtaatcatggtca-3') and an arbitrary degenerate primer (5'-ngtcgaswganawgaa-3') were used for TAIL-PCR amplification. The thermal cycling conditions are summarised in Table 1. Briefly, the primary PCR reaction contained 0.5-1.0 μg genomic DNA, 1.5 mM dNTPs, 0.2 μM of tgp1 primer, 2 μM of the a.d. primer, and 1 unit of T*aq *polymerase in 20 μl of 1 × reaction buffer. In the secondary or tertiary amplification, 0.65 μl of the first or secondary products, respectively, were used as templates and supplemented in the reaction. The products of tertiary TAIL-PCR reaction were separated on a 1.5% agarose gel. A single band in each mouse sample was gel purified with the TIANgel Midi Purification Kit (Tiangen, China) and sequenced directly. The resultant sequences were analysed using online BLAST from the database of *Mus musculus *genomic DNA http://www.ncbi.nlm.nih.gov.

**Table 1:** Cycle Conditions used for TAIL-PCR

Reaction	Program **no**.	**Cycle no**.	Thermal condition
Primary (tgp1/ad)	1	1	95°C, 5 min
	2	10	94°C, 10 sec; 63°C, 30 sec; 72°C, 3 min
	3	1	94°C, 10 sec; 25°C, 3 min; 72°C, 2.5 min
	4	15	94°C, 10 sec; 63°C, 3 min; 72°C, 2.5 min; 94°C, 10 sec; 63°C, 3 min; 72°C, 2.5 min; 94°C, 10 sec; 44°C, 1 min; 72°C, 2.5 min
Secondary (tgp2/ad)	5	1	95°C, 5 min
	6	15	94°C, 10 sec; 65°C, 3 min; 72°C, 2.5 min; 94°C, 10 sec; 65°C, 3 min; 72°C, 2.5 min; 94°C, 10 sec; 44°C, 1 min; 72°C, 2.5 min
Tertiary (tgp3/ad)	5	1	95°C, 5 min
	6	15	94°C, 10 sec; 65°C, 3 min; 72°C, 2.5 min; 94°C, 10 sec; 65°C, 3 min; 72°C, 2.5 min; 94°C, 10 sec; 44°C, 1 min; 72°C, 2.5 min

### PCR analysis of transgene homozygotes and heterozygotes

Genomic DNA from the tails of C57 females (wild type), *fad2 *males (X^+^Y), or homozygous (X^+^X^+^) or heterozygous (X^+^X) *fad2 *females was used to determine their homozygosity or heterozygosity. In brief, genome-specific primers for 495-bp fragments (F: 5'-agtctgcaattttagatcctc-3'; R: 5'-gaagtttcagcagcaacac-3') and transgene-specific primers for 567-bp fragments were added to the same PCR reaction and amplified together. The PCR cycling parameters were as follows: 95°C for 5 min; 35 cycles of 94°C for 30 sec, 56°C for 30 sec, and 72°C for 60 sec; and 72°C for 10 min.

### PCR analysis of the 5' integration site

Based on the results of the 3' integration analysis, each DNA sample of X^+^Y, X^+^X^+^, X^+^X or C57 mice was amplified by the following primer sets: P1 (F: 5'-cgctcagtcagtcaccag-3'; R: 5'-ggacttcgattaccgttt-3'), P2 (F: 5'-catcagtgctcccattct-3'; R: 5'-aacaggcttgtggtcatt-3'), and P3 (F: 5'-aactcaagggacaccaca-3'; R: 5'-aatccgcatgaataccag-3'). The three primer pairs corresponded to the upstream 3' integration regions from -54,088 to -53,884, -29,872 to -29,553, and -44,562 to -44,144, respectively. PCR amplification was performed as follows: 95°C for 5 min; 30 cycles of 94°C for 30 sec, 57.6°C for 30 sec, and 72°C for 60 sec; and a final extension at 72°C for 10 min. P4 forward primer at -46,680 (5'-agcctagtggtacatcat-3') or P5 forward primer at -45,797 (5'-ggtatcttgttccctattc-3') of the 3' integration upstream region was used along with the common reverse primer (5'-gccaagtgggcagttta-3'), corresponding to the CMV molecules needed to amplify the relevant junction sequences. P4 amplification was performed as follows: 95°C for 5 min; 40 cycles of 94°C for 30 sec, 56.4°C for 30 sec, and 72°C for 2 min; and a final extension at 72°C for 8 min. P5 conditions were: 40 cycles of 94°C for 30 sec, 58°C for 30 sec, and 72°C for 60 sec. P4 and P5 products were sequenced directly.

### RT-PCR

Total RNA was extracted from the fresh tissues (liver, kidney, brain, muscle and heart) of *fad2 *or C57 females with TRIzol Reagent (Tiangen) and treated with RNase-free DNase I (TaKaRa) to remove the remaining genomic DNA prior to RT-PCR. Purified RNA (0.5 μg) was used for first-strand cDNA synthesis. Reverse transcription was performed using M-MLV reverse transcriptase (Promega) with oligo-dT primers according to the manufacturer's instructions. Reactions in the absence of reverse transcriptase were also included for each RNA sample to check for DNA contamination. The resultant cDNA was used in PCR amplification to investigate the level of gene transcription. Besides the transcriptional amplification of the *fad2 *gene using transgene-specific primers (F: 5'-tacatcagcgacacaggcatc-3'; R: 5'-gtatttgtgagccagggcatt-3'), RT-PCR was also used to investigate the expression of the 5' integration upstream region from -1123 to -977 (F: 5'-agcctagtggtacatcat-3'; R: 5'-ttggcctacattagacat-3') and the 3' integration downstream region from +859 to +1166 (F: 5'-attaggtcccctcagtgtc-3'; R: 5'-ctcatctcagaaatcattaccc-3'), +1952 to +2299 (F: 5'-tgacagagcgtctaagga-3'; R: 5'-gaggtaacccaatcacaaa-3'), and +1952 to +3069 (F: 5'- tgacagagcgtctaagga-3'; R: 5'-cagaacaccaatggcttg-3'). The annealing temperature was 55°C, 55°C, 56°C, or 57.6°C, respectively. Concurrently, the 352-bp control fragments of the *Hprt *gene (F: 5'-cctgctggattacattaaagcactg-3'; R: 5'-gtcaagggcatatccaacaacaaac-3') were amplified as follows: 95°C for 5 min; 35 cycles of 94°C for 30 sec, 55°C for 30 sec, and 72°C for 60 sec; and final extension at 72°C for 8 min.

### Real-time RT-PCR

Real-time RT-PCR was used to determine the relative expression of the *fad2 *transgene (F: 5'-ttccacaacatcaccgacac-3'; R: 5'-ctccacgtacaggcactcc-3') in transgenic kidney, brain and liver tissues. *Gapdh *(F: 5'-gaacatcatccctgcatcc-3'; R: 5'-ccagtgagcttcccgttca-3') was amplified concurrently as an endogenous control. Each sample from X^+^X^+^, X^+^X, C57 females or X^+^Y males was amplified in triplicate. Real-time PCR reactions were performed on an ABI Prism 7500 sequence detection system (Applied Biosystems) using SYBR^® ^Premix Ex Taq™ II kits (TaKaRa), following the manufacturer's protocol. The thermocycling protocol was as follows: 95°C for 30 sec, 40 cycles of 95°C for 5 sec and 60°C for 34 sec. Expression of the *fad2 *gene was examined relative to the internal control gene using the 2^(-ΔΔC(T)) ^method.

### Statistical analysis

RNAs from two mice of each genotype were processed and amplified by real-time RT-PCR, with at least three measurements per animal. All values are presented as means ± SEM. A ratio was considered significant if the mean *t*-test *P *value was less than or equal to 0.05 for each of the samples. Prism 4 for Windows (GraphPad) was used for all calculations.

## Authors' contributions

BY carried out the molecular studies and data analysis. DL participated in the design of the study and assisted in writing the manuscript. KG conceived of the study, and participated in its design and coordination and wrote the manuscript. All authors read and approved the final manuscript.
